# Analysis of landscape pattern vulnerability in Dasi river basin at the optimal scale

**DOI:** 10.1038/s41598-024-61634-x

**Published:** 2024-05-10

**Authors:** Haocheng Wang, Lin Wang, Xia Liu, Baoli Wei

**Affiliations:** 1 College of Environmental Science and Engineering, Ocean University of China, Qingdao, 266100 China; 2 Qingdao City South District Comprehensive Administrative Law Enforcement Bureau, Qingdao, 266000 China; 3 Survey and Mapping Institute of Qingdao City, Qingdao, 266100 China

**Keywords:** Ecology, Environmental sciences, Environmental social sciences

## Abstract

Since the reform and opening up in 1978, the Dasi River Basin within Jinan’s startup area from replacing old growth drivers with new ones (startup area) has experienced rapid urbanization and industrialization, and the landscape pattern has changed significantly, resulting in a series of eco-environmental problems. In order to more accurately identify the vulnerable areas of landscape pattern, understand their cause mechanism and changing laws, and provide a theoretical basis for the implementation of sustainable landscape pattern planning and management in the region. Four Landsat images of 2002, 2009, 2015 and 2020 were taken as data sources, and the optimal granularity of landscape pattern analysis was determined from the perspective of landscape level and class level by using the coefficient of variation method, granularity effect curve and information loss model, and the optimal amplitude was determined by using the grid method and semi-variance function. Then, the landscape vulnerability assessment model was constructed based on the optimal scale, and its spatiotemporal evolution characteristics and spatial autocorrelation were analyzed. The result showed that: (1) The optimal granularity of landscape pattern analysis in this study area was 80 m, and the optimal amplitude was 350 × 350 m. (2) During 2002–2020, the overall vulnerability of landscape pattern in the southern part of the study area showed an increasing trend, while that in the middle and northern parts showed a decreasing trend. (3) The mean values of the vulnerability index of the overall landscape pattern in 2002, 2009, 2015 and 2020 were 0.1479, 0.1483, 0.1562 and 0.1625, respectively, showing an increasing trend year by year. In terms of land use, during 2002–2020, the average vulnerability indices of forestland and built up land increased by 23.18% and 21.43%, respectively, followed by water body and bare land, increased by 12.18% and 9.52%, respectively, while the changes of cropland and grassland were relatively small, increasing by 5.36% and 5.65%, respectively. (4) During 2002–2020, the landscape pattern vulnerability showed a significant spatial positive correlation in terms of spatial distribution. The Low-Low areas were generally transferred from the southeastern and midwestern to the middle and northern, and the High–High areas were mainly transferred from the middle to the southern. Overall, the degree of the spatial agglomeration of the landscape pattern vulnerability showed an increasing trend.

## Introduction

Landscape pattern refers to the spatial arrangement and combination of various landscape patches in different shapes and sizes, which is the result of the interaction between human activities and the natural environment on the earth surface^1,2^. Landscape pattern is closely related to ecological processes^3,4^. Changes in landscape pattern will have a direct impact on the stability, anti-interference ability, material circulation and energy flow, as well as ecological service functions of ecosystems^5,6^. Since the mid-twentieth century, driven by population and economic interests, the frequency and intensity of land use landscape pattern changes have increased significantly, and the pressure and risk of ecological environment have increased accordingly^7–9^. A series of ecological vulnerability problems such as urban heat island effect^10^, water shortage^11^, water quality degradation^12^, land degradation^13^, flood disaster^14^ and biodiversity reduction^15^ have been generated, which greatly limit the sustainable development of landscape pattern^16^. In this context, it is particularly important to conduct research on the vulnerability of landscape pattern.

Recently, there have been many researches on the characteristics of landscape pattern change. For example, Alaei^17^ analyzed the spatial differences in landscape fragmentation levels at different scales in the Koozeh Topraghi Watershed. Mostafazadeh^18^ analyzed the changing characteristics of land use landscape patterns in the mountainous areas of northwestern Iran using 20 landscape indices and predicted future land use patterns. Abdolalizadeh^19^ analyzed the evolution characteristics of landscape pattern in Marakan protected area during 1986–2016 by selecting 10 landscape indices at the landscape and class levels. Xu^20^ analyzed the spatial–temporal evolution characteristics of landscape ecological risk under multiple scenarios in Xinjiang based on the Markov-FLUS composite model. Mostafazadeh^21^ analyzed the spatial distribution characteristics of ecological security in the Koozeh Topraghi Watershed using the landscape ecological security index. However, the research on landscape pattern vulnerability is relatively rare. Landscape pattern vulnerability originates from ecological vulnerability, and is an attribute that reflects the sensitivity of landscape patterns to external disturbances (natural environment and human activities) and the adaptability to maintain the structure, function, and characteristics of landscape systems unchanged^22–24^. It emphasizes the establishment of the relationship between pattern information and ecological vulnerability from the perspective of landscape ecology^22,25,26^. The landscape pattern index can highly concentrate information, effectively representing the landscape pattern characteristics such as landscape change, structural composition and spatial configuration^27,28^. Therefore, many scholars have attempted to use various landscape indices with different ecological significance to construct a landscape pattern vulnerability assessment framework to measure ecosystem stability. For example, Ortega^29^ evaluated the vulnerability of Spanish rural landscapes to wildfires by selecting 12 landscape indices that reflect landscape composition and configuration characteristics. Zang^23^ established the response relationship between landscape pattern and ecological vulnerability by using landscape indices including patch number, patch density, average patch size and class area. Zhou^30^ constructed a landscape pattern vulnerability assessment framework using landscape sensitivity index and landscape adaptive index, and analyzed the impact of human activities on landscape vulnerability in the coastal areas of Jiangsu Province from 2000 to 2015. Yu^31^ explored the spatiotemporal changes and influencing factors of landscape ecological vulnerability in the Three-River-Source National Park Region from 1995 to 2015. Taking Hengduan Mountain range as the research object, sun^32^ established a landscape pattern vulnerability assessment framework and analyzed its response relationship with terrain and human-induced disturbance intensity. Wang^16^ proposed landscape pattern optimization suggestions based on the spatiotemporal changes of landscape pattern vulnerability in Wuhan metropolitan area from 2000 to 2020. This method gives full play to the characteristics of remote sensing (RS) and geographic information system (GIS), such as easy to obtain data, long-term and large-scale, and better applicability even in areas lacking historical ecological monitoring data^33,34^. It has become an essential method in the study of ecological vulnerability assessment^32^.

Landscape patterns display typical spatial heterogeneity and scale dependence, and these are fundamental characteristics of spatial heterogeneity, as reflected in granularity and amplitude effects^35,36^. In recent years, more and more researchers have begun to pay attention to the impact of scale effects on landscape pattern characteristics. For example, Zhang^37^ proposed that the optimal granularity range for analyzing the landscape pattern changes in the Three Gorges Reservoir was 30-60 m. Li^38^ found that the optimal amplitude for analyzing landscape pattern characteristics in the Chinese coastal zones was 5 km. Ju^39^ determined that the optimal amplitude for landscape ecological risk analysis in the Shandong Peninsula was 4 km. Ai^36^ determined that the optimal granularity for landscape ecological risk analysis in the Haitan Island was 75 m. Although there are many studies on the scale effects of landscape patterns, the choice of scale often depends on personal experience or only considers the granularity or amplitude, which makes it less convincing in describing landscape pattern characteristics^36^. In terms of assessing the vulnerability of landscape patterns, most scholars only focus on the exploration of its spatiotemporal evolution laws, ignoring the impact of scale effects on its spatial heterogeneity, which has a certain negative impact on the scientific and accurate of the assessment results^40^. Therefore, it is necessary to determine the optimal granularity and amplitude of landscape pattern analysis before conducting landscape pattern vulnerability assessment.

The startup area is the second demonstration area after the Xiongan new area in China, and is also the strategic deployment of implementing ecological protection and high-quality development in the lower reaches of the Yellow River basin^41^. This study area is located in the central zone of the startup area, and it is also one of the key projects of the planning and construction of the startup area in the future. This means that the future landscape pattern changes in this area will be more complicated. How to coordinate the balance between economic development and ecological protection is a key issue for the local government to formulate national economic development goals. Taking Dasi river basin within the startup area as the object, starting from exploring the optimal scale of landscape pattern, a landscape vulnerability evaluation model based on landscape pattern index is established to obtain more scientific and accurate spatiotemporal change information of landscape pattern vulnerability. According to the evaluation results, decision-makers can effectively identify the vulnerable areas of the ecological environment, understand their cause mechanism and changing laws. It has certain practical significance and application value to guide the rational development of national spatial planning and landscape pattern optimization in this region, and realize the win–win goal of regional economic development and ecological protection.

**Figure 1:**
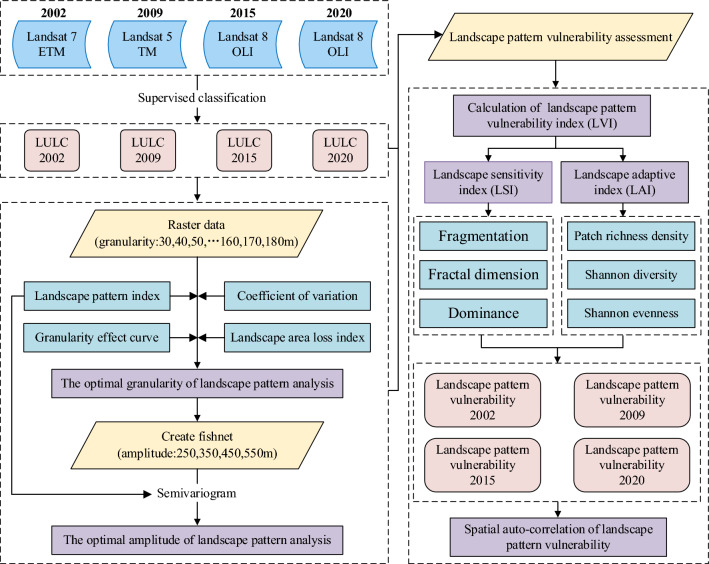
The framework of this study.

Overall, this paper has three aims: (1) to identify the landscape pattern indices with high sensitivity to granularity changes using the coefficient of variation method; (2) to determine the optimal granularity and amplitude of landscape pattern analysis in the study area using the granularity effect curve of high sensitivity landscape indices, area information loss index, and Semivariogram; (3) to explore the spatiotemporal evolution patterns and spatial aggregation characteristics of landscape pattern vulnerability. The analysis framework of landscape pattern vulnerability based on optimal scale is presented in Fig. 1.

## Study area and data

### Study area

**Figure 2:**
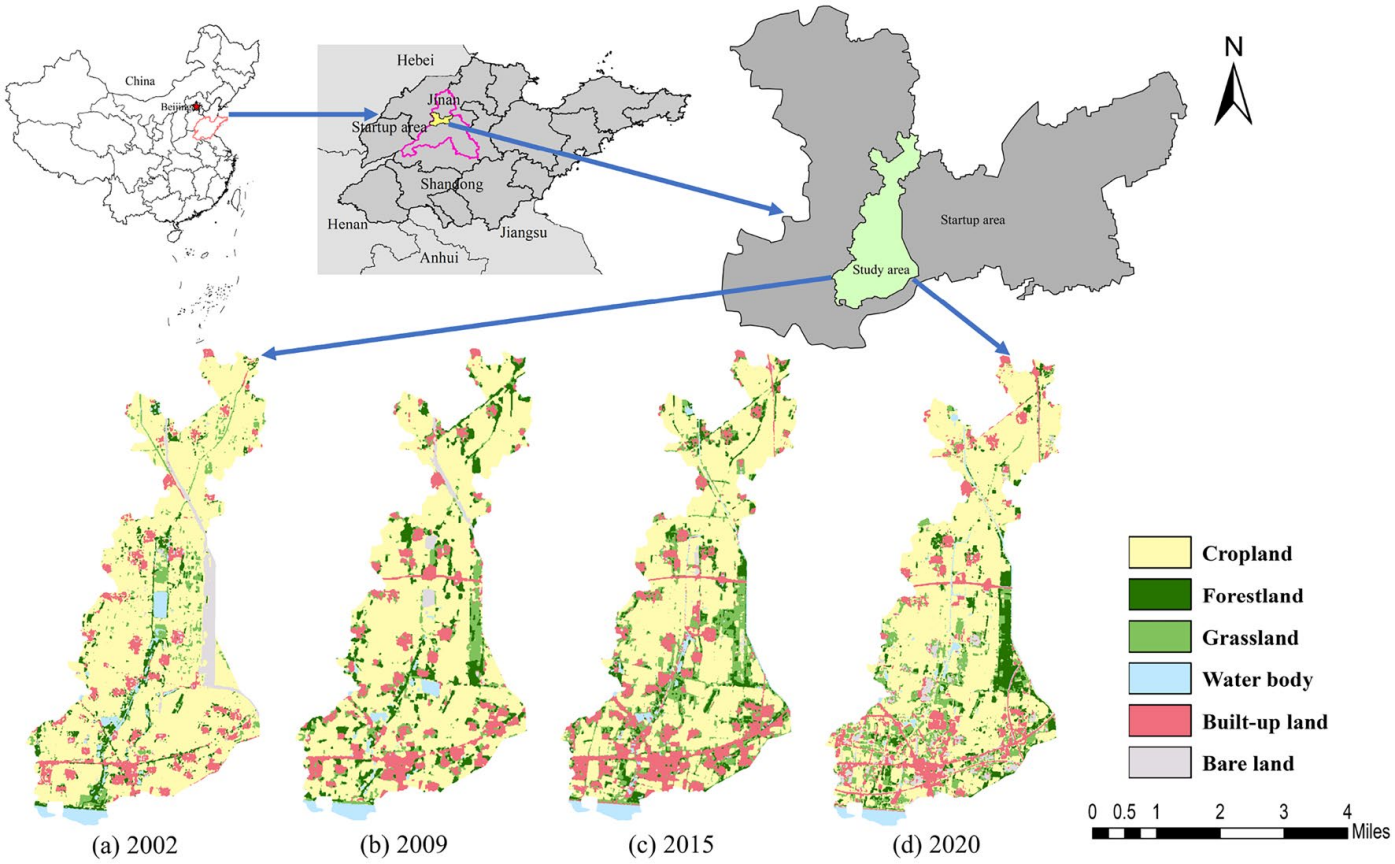
Geographical location and land use types of the study area. Cartographic software: ArcGIS online (https://www.esri.com/en-us/home).

Jinan is the capital of Shandong Province, and also an important city in northern China and the Yellow River basin. In order to promote the high-quality development of urban agglomeration in Shandong Peninsula, the Chinese government has decided to carry out the construction of the startup area. The startup area is located in the northern part of Jinan, west to Dezhou, east to the Xiaoqing River, south to the Yellow River, north to the Tuhai River, with an area of about 798 km^2^. At the end of 2021, the total population was about 425,000, and the regional GDP was about 6.11 billion yuan. It is estimated that by 2030, the total population will reach 1.1 million and the regional GDP will reach 160 billion yuan. The Dasi River originates from the Queshan Reservoir and flows into the Tuhai River from south to north and plays an important role in flood control, drainage and irrigation. The study area is located in the middle of the startup area with an area of 75.47 km^2^. Previous studies have shown that the cropland is the main land use type in the study area, and the land use pattern has changed significantly during the past 20 years, leding to a certain degree of degradation of the ecological service functions provided by the ecosystem^41^. The climate is characterized by hot summer and cold winter, belonging to the north warm temperate monsoon climate. The annual average precipitation is about 614 mm, with over 60% concentrated in summer. Throughout the year, the wind is predominantly from the south-west, followed by the north-east, with an annual average wind speed of 1.9 m/s. The annual average temperature and total sunshine hours are about 13.6 °C and 2347.10 h, respectively. Mineral resources are abundant, containing a large amount of non-ferrous metals such as iron, coal, copper and potassium. Grain crops are mainly rice, wheat, corn, and soybean. In addition, there are a variety of economic crops, poultry, livestock, and aquatic products. The geographical location and land use types of the study area are shown in Fig. 2, which are prepared by using ArcGIS 10.6 (ESRI, Redlands, CA, USA).

### Data sources and pre-processing

Four remote sensing images including the Landsat 7 ETM + image of October 6, 2002; the Landsat 5 TM image of June 11, 2009; and the Landsat 8 OLI/TIRS images of June 12, 2015, and May 27, 2020, were obtained from the geospatial data cloud (http://www.gscloud.cn/) at a spatial resolution of 30 m. Then, a series of preprocessing such as radiometric calibration, atmospheric correction, projection transformation and region clipping were carried out by using ENVI 5.3 (ITT Visual Information Solutions, Boulder, CO, USA). According to the research objectives and the characteristics of land use classification of China, the land use types of the study area were divided into 6 categories using ArcGIS 10.6 (ESRI, Redlands, CA, USA), including, cropland, forestland, grassland, water body, built-up land, and bare land (Fig. 2). Compared with the Google Earth images in the corresponding period, the land use classification results are highly consistent with the actual land use. The classification accuracy of land use in 2002, 2009, 2015 and 2020 were 87.85%,88.15%, 86.24% and 86.73%, respectively. Therefore, the obtained land use raster data can be used for subsequent research on landscape pattern vulnerability.

## Methods

### Spatial effect analysis

#### Granularity effect

1. Determination of sensitive landscape pattern index.

The landscape pattern index can quantitatively describe the spatial structure characteristics of the whole landscape or specific landscape elements, which is divided into three levels: patch, class and landscape^42,43,44^. Commonly, the selection of suitable landscape metrics involves using correlation analysis or principal component analysis (PCA) to identify the optimal landscape metrics. In order to identify the optimal granularity of landscape pattern analysis, it is necessary to select the landscape pattern index which is sensitive to the change of granularity. Firstly, by using the sampling tool in ArcGIS 10.6, the land use maps of the four periods were converted into raster data with different granularities of 30, 40, 50, 60, 70, 80, 90, 100, 110, 120, 130, 140, 150, 160, 170 and180 m. Then, we followed the principle of wide-ranging but not redundant to select landscape pattern indices, including 12 class level indices and 17 landscape level indices, and classified them into four categories: area and edge, shape, aggregation, and diversity metrics (Table 1). Based on the generated land use maps with different resolutions, these selected landscape pattern indices were calculated by using Fragstats 4.3 software^45^.

**Table 1:** Selected landscape indices at the class and landscape levels.

Category	Item	Abbr	Unit	Class level	Landscape level
Area and edge	Edge density	ED	m/ha	Yes	Yes
	Number of patch	NP	#	Yes	Yes
	Patch density	PD	#/100 ha	Yes	Yes
	Largest patch index	LPI	%	Yes	Yes
	Mean patch area	AREA_MN	ha	Yes	Yes
Shape	Perimeter-Area Fractal Dimension	PAFRAC	—	Yes	Yes
	Mean shape index	SHAPE_MN	—	Yes	Yes
	Mean Fractal dimension index	FRAC_MN	—	Yes	Yes
Aggregation	Mean Euclidean Nearest-Neighbor Distance	ENN_MN	m	Yes	Yes
	Percentage of Like Adjacencies	PLADJ	%	Yes	Yes
	Interspersion and Juxtaposition Index	IJI	%	Yes	Yes
	Patch Cohesion Index	COHESION	—	Yes	Yes
	Effective mesh size	MESH	ha	No	Yes
	Contiguity index	CONTAG	%	No	Yes
Diversity	Patch Richness Density	PRD	—	No	Yes
	Shannon’s diversity index	SHDI	—	No	Yes
	Shannon’s evenness index	SHEI	—	No	Yes

The coefficient of variation (CV) is a statistic used to measure the relative statistical dispersion of sequence eigenvalues, which is expressed as the ratio of the standard deviation and the average value of the data^46^. It reflects the sensitivity of the measured indices to granularity changes, and the larger the value, the more sensitive the index is to granularity change. The specific formula is as follows^46^:

1 $$\mathop C\nolimits_{{\text{v}}} = \frac{1}{{\overline{V} }}\sqrt {\frac{{\sum\limits_{i = 1}^{m} {\left( {\mathop V\nolimits_{i} - \overline{V} } \right)^{2} } }}{m - 1}} \times 100{\text{\% }}$$

where *V*_*i*_ is the value of a certain landscape index at the *ith* granularity; *V* is the average value of a landscape index at all granularities; *m* is the number of granularity set.

(2) Inflection point analysis method.

According to the results of coefficient of variation analysis, the land use map of 2020 was selected as the sample, and the granularity effect curves of high sensitivity landscape pattern indices at the landscape level and the class level were plotted respectively. Considering that the landscape pattern indices change greatly near the inflection point, the inflection point analysis method was used to determine the change trend of high sensitivity landscape pattern indices with different granularities. The first granularity interval with smaller changes was selected as their respective suitable granularity domain, and their intersection was selected as the optimal granularity domain for the regional landscape pattern research^47^.

(3) Information loss evaluation model.

In order to make up for the limitation of determining the optimal granularity domain only based on the granularity effect curve, an information loss assessment model was introduced into this study. By comparing the landscape area under different granularities after conversion with the landscape area of vector data before conversion, the relative values of landscape area loss under different granularities after conversion were obtained. The smaller the value, the higher precision of data conversion under this granularity. The information loss evaluation model is as follows^48^:

2 $$\mathop L\nolimits_{{\text{i}}} = \left( {\mathop A\nolimits_{i} - \mathop A\nolimits_{hi} } \right)/\mathop A\nolimits_{hi} \times 100{\text{\% }}$$

3 $$S = \sqrt {\frac{{\sum\limits_{i = 1}^{n} {\mathop L\nolimits_{i}^{2} } }}{n}}$$

where *L*_*i*_ refers to the relative value of landscape area loss, *n* is the total number of landscape types, *S* refers to the total value of landscape area loss, and *A*_*i*_ and *A*_*hi*_ refer to the area of the *ith *landscape type after and before conversion, respectively.

#### Semivariogram

Based on the land use map at the optimal granularity, several square units with different amplitudes were created by using the fishnet tool in ArcGIS 10.6. Each unit was taken as a statistical sample under the spatial amplitude^36^. By calculating the landscape pattern index in each unit and assigning it to the corresponding unit center, the grid map of landscape pattern index under the different amplitudes can be obtained. Considering the actual area and workload of the study area, we set four amplitude ranges, including 250 × 250, 350 × 350, 450 × 450 and 550 × 550 m. The numbers of the statistical units were 1281, 667, 423, and 286. As a function of variance and distance of data points in geostatistics, the semivariogram can effectively reveal the spatial heterogeneity of variables, and the optimal spatial amplitude can be explored by fitting the results of the semivariogram to the landscape pattern indices with different amplitudes^49^. The calculation formula for the semivariogram (*γ*(*h*)) is as follows^36^:

4 $$\gamma \left( {\text{h}} \right) = \frac{1}{2N\left( h \right)}\sum\limits_{i = 1}^{N\left( h \right)} {\left[ {Z\left( {\mathop x\nolimits_{i} } \right) - Z\left( {\mathop x\nolimits_{i} + h} \right)} \right]^{2} }$$

where *N*(*h*) is the number of data pairs separated by lag *h*, and *Z*(*x*_*i*_) and *Z*(*x*_*i*_ + *h*) denote the values of the regionalized random variables *X*_*i*_ and *X*_*i*_ + *h*, respectively. ArcGIS Online software is used for map making. See https://www.esri.com/en-us/home for details.

### Assessment of landscape pattern vulnerability

Referring to the relevant literature^30^, we used landscape sensitivity index (LSI) and landscape adaptive index (LAI) to construct the landscape pattern vulnerability index (LVI), so as to carry out quantitative research and visual expression of landscape pattern vulnerability in the study area.

#### Construction of landscape sensitivity index

Different landscape types played different roles in maintaining species diversity, ensuring the normal operation of ecological flow, energy flow and functional flow in the system, and promoting the natural succession of landscape structure. At the same time, there were differences in the anti-interference ability of different landscape types to the external environment^50^. Therefore, landscape disturbance index and landscape type vulnerability index were selected to construct the landscape sensitivity index. The former reflects the degree of external disturbance to the landscape, while the latter reflects the degree of landscape loss to disturbance^32^. The formula is as follows:

5 $$LSI_{k} = \sum\limits_{i = 1}^{n} {\mathop U\nolimits_{ki} \times \mathop V\nolimits_{i} }$$

where *k* is the grid unit; *i* represents the *ith* landscape type; *N* is the number of landscape types; *LSI*_*k*_ is the landscape sensitivity index of the *kth* grid unit; *U*_*ki*_ is the landscape disturbance index of *ith* landscape type in the *kth* grid unit; *V*_*i*_ is the degree of vulnerability of *ith* landscape type. Bare land and grassland were the most sensitive to external disturbance, so the vulnerability assigned to bare land and grassland were 6 and 5 respectively. The economic value of forestland was high, so a value of 4 was assigned to forestland. The value of cropland was 3. As an important resource supporting human production and life, water body has a relatively low possibility of change under external interference, so the value of water body was 2. The landscape structure of built-up land was the most stable, so the vulnerability assigned to built-up land was 1. After normalization, the vulnerability values of the six landscape types were as follows: bare land, 0.2857, grassland, 0.2381, forestland, 0.1905, cropland, 0.1429, water body, 0.0952 and built-up land, 0.0476.

The LSI is mainly composed of three landscape pattern indices: fragmentation index (FN), fractal dimension (FD), and dominance index (DO). The formula is as follows^32^:

6 $$U_{ki} = aFN_{ki} + bFD_{ki} + cDO_{ki}$$

$$FN_{ki} = \frac{{N_{ki} }}{{A_{ki} }}$$

7 $$FD_{ki} { = }\left[ {2 \times ln\left( {P_{ki} /4} \right)} \right]{/}ln\left( {A_{ki} } \right)$$

8 $$DO_{ki} = 0.4 \times \frac{{N_{ki} }}{{N_{k} }} + 0.6 \times \frac{{A_{ki} }}{{A_{k} }}$$

where, *FN*_*ki*_ is the fragmentation index of the *ith* landscape type in the *kth* grid unit, *FD*_*ki*_ is the fractional dimension of the *ith* landscape type in the *kth* grid unit, and *DO*_*ki*_ is the dominance index of the* ith* landscape type in the* kth* grid unit. The weights of the three indices (a, b, and c) were assigned as 0.5, 0.3, and 0.2, respectively.

#### Construction of landscape adaptive index

The vulnerability of landscape pattern was not only affected by sensitivity, but also related to the adaptability of landscape system. Generally, a more complex structure and uniform distribution indicate a more stable system. So, three representative landscape pattern indices, including the patch richness density index (PRD), the Shannon diversity index (SHDI), and the Shannon evenness index (SHEI) were selected to construct the LAI. The formula is as follows^31^:

9 $$LAI_{k} = PRD_{k} \times SHDI_{k} \times SHEI_{k}$$

#### Construction of landscape pattern vulnerability index

According to the connotation of landscape vulnerability, landscape vulnerability is directly proportional to landscape sensitivity and inversely proportional to landscape adaptability. The specific calculation formula is as follows^31^:

10 $$LVI_{k} { = }LSI_{k} \times \left( {1 - LAI_{k} } \right)$$

### Spatial auto‑correlation analysis

Spatial autocorrelation is a common method to measure the spatial correlation between an eigenvalue and those in its adjacent spatial unit^51^. In order to explore the spatial correlation of the landscape vulnerability index of each grid unit in the whole region and the spatial correlation of the landscape vulnerability index of a grid unit and its adjacent spatial units, we analyzed the spatial distribution characteristics of the landscape pattern vulnerability from the perspective of global spatial autocorrelation and local spatial autocorrelation respectively. The Moran’s I scatter graph and LISA cluster map, which reflect the spatial correlation of LVI, are both prepared by using GeoDa1.18 software.

#### Global spatial auto‑correlation

Global spatial autocorrelation can reflect whether a certain eigenvalue has spatial aggregation in the whole region, which is expressed by the global Moran’s I. The formula for the global Moran’s I index is as follows^52^:

11 $$I = \frac{{n\sum\nolimits_{i = 1}^{n} {\sum\nolimits_{{j{ = }1}}^{n} {W_{ij} \left( {x_{i} - \overline{x} } \right)} } \left( {x_{j} - \overline{x} } \right)}}{{\sum\nolimits_{i = 1}^{n} {\left( {x_{i} - \overline{x} } \right)}^{2} \sum\nolimits_{i = 1}^{n} {\sum\nolimits_{{j{ = }1}}^{n} {W_{ij} } } }}$$

where *I* is the global Moran’s I, range from + 1 to − 1. When the value is close to 1, it means that the positive correlation of the LVI of each grid unit in the study area is stronger, and the spatial distribution is clustered. When the value is close to − 1, the stronger the negative correlation is, and the spatial distribution is discrete. When the value is 0, there is no correlation and the spatial distribution is random; *x*_*i*_ and *x*_*j*_ are the eigenvalues of the *ith* and *jth* grid units, respectively; *W*_*ij*_ is the spatial weight between unit *i* and* j*, *‾x* is the mean value of eigenvalues; *n* is the total number of grid units.

#### Local spatial auto‑correlation

Local spatial autocorrelation can reflect the spatial heterogeneity of an eigenvalue in a local spatial unit and its adjacent spatial units, which is expressed by the local indicator of spatial association (LISA). The calculation formula is as follows^53^:

12 $$LISA_{i} = \frac{{n\left( {x_{i} - \overline{x} } \right)}}{{\sum\nolimits_{i = 1}^{n} {\left( {x_{i} - \overline{x} } \right)^{2} } }}\sum\nolimits_{j = 1}^{n} {w_{ij} \left( {x_{j} - \overline{x} } \right)}$$

## Results

### Spatial effect analysis of landscape pattern index

#### Granularity effect analysis of landscape pattern index

1. Granularity effect analysis of landscape level indices.

The CV of the landscape pattern index reflects the degree of response to the increase in granularity, and it was divided into 4 sensitivity levels in our research: high sensitivity (CV > 20%), moderate sensitivity (20% > CV > 10%), low sensitivity (10% > CV > 1%) and insensitivity(CV < 1%).The CV results of the indices measured at the landscape level are shown in Table 2.

**Table 2:** The CV results of the indices measured at the landscape level (%).

Landscape pattern index	2002	2009	2015	2020
NP	70.84	51.59	71.33	75.91
PD	70.81	51.53	71.30	75.89
LPI	11.10	24.87	19.33	23.89
ED	33.36	23.62	31.79	35.45
AREA_MN	52.66	46.31	54.06	58.61
SHAPE_MN	2.90	3.45	2.58	2.37
FRAC_MN	0.72	0.88	0.71	0.65
PAFRAC	2.65	4.32	3.04	3.59
ENN_MN	42.42	39.72	44.66	45.42
CONTAG	6.01	9.87	10.71	8.90
PLADJ	8.83	12.63	13.14	11.66
IJI	1.50	1.32	0.91	0.87
COHESION	0.77	0.88	1.79	1.10
MESH	18.40	39.17	30.43	28.13
PRD	0.32	0.32	0.32	0.32
SHDI	0.95	0.69	0.38	0.62
SHEI	0.96	0.69	0.38	0.62

**Figure 3:**
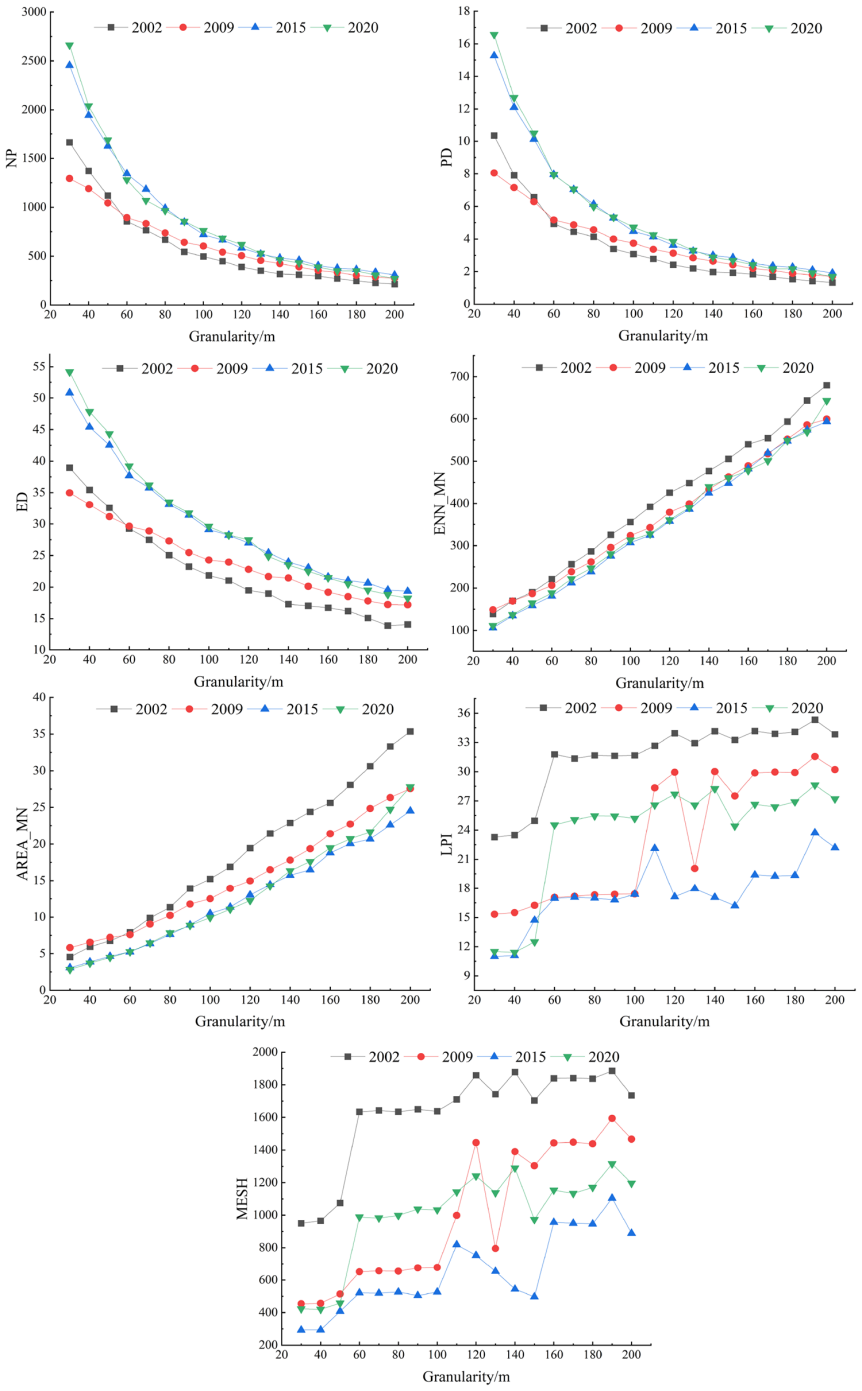
The granularity effect curves of high sensitivity landscape pattern indices at the landscape level during 2002–2020.

As shown in Table 2, in 2020, the number of extremely high and highly sensitive indices was the largest and the corresponding coefficient of variation was also the largest. Therefore, the land use map of 2020 was selected to analyze the granularity effect of landscape pattern index. The indices at the landscape level with high sensitivity were NP, PD, ED, AREA_MN, ENN_MN, LPI, and MESH. Within the set granularity range, these indices changed significantly with the increase of granularity. The only index with moderate sensitivity was PLADJ, which was associated with moderate change when granularity increase. The indices with low sensitivity were SHAPE_MN, PAFRAC, CONTAG and COHESION, which responded to a change within a small range. FRAC_MN, IJI, PRD, SHDI and SHEI were insensitive indices, which did not change significantly with the increase of granularity. The granularity effect curves of high sensitivity landscape pattern indices at the landscape level were shown in Fig. 3.

The landscape pattern index selected at the landscape level showed different trends with the increase of granularity (Fig. 3). According to the characteristics of the granularity effect curve, it was divided into three categories. The first category included NP, PD and ED. These landscape pattern indices decreased rapidly first and then decreased slowly with the increased of granularity. The second category included AREA_MN and ENN_MN, which was characterized by a roughly linear increased in landscape pattern indices with the increased of granularity. The third category included LPI and MESH, which was characterized by that with the increased of granularity, the landscape indices first remained stable and then changed sharply, and then returned to stable. However, when the granularity increased to a certain extent, the landscape pattern indices fluctuated sharply.

As shown in Fig. 3, when the granularity reached 60 m, the decreasing trend of NP, PD and ED changed from fast to slow, so 60 m could be used as its ' inflection point '. ENN _ MN and AREA _ MN basically maintain a certain slope increased, which could be considered as no ‘inflection point’. LPI and MESH had obvious ‘inflection points’ at 40 m, 60 m and 100 m. When the granularity range was between 30–40 m, 60–100 m and 160–180 m, the landscape pattern indices were in a relatively stable state, while in other granularity ranges, the landscape pattern indices were in a state of obvious fluctuation. Therefore, 60–100 m was determined as the suitable granularity range of the whole landscape in the study area.

(2) Granularity effect analysis of class level indices.

In order to ensure the accuracy of the research results, the response of the granularity change of the landscape pattern index at the class level should also be considered. Consistent with the research idea of landscape level, taking the land use map of 2020 as an example, the CV of the landscape pattern index selected at the class level was calculated. The calculation results are shown in Table 3.

**Table 3:** The CV results of the indices measured at the class level (%).

Landscape pattern index	Cropland	Forestland	Grassland	Water body	Built up land	Bare land
NP	113.98	76.13	76.38	72.47	61.37	55.68
PD	114.00	76.11	76.37	72.44	61.33	55.62
LPI	23.89	14.76	33.56	4.88	17.21	13.74
ED	34.64	37.21	37.92	42.60	32.74	30.93
AREA_MN	79.88	58.34	60.04	61.43	50.45	50.42
SHAPE_MN	10.75	2.38	3.03	4.73	3.33	5.07
FRAC_MN	0.71	0.64	0.72	0.96	0.65	1.02
PAFRAC	3.83	4.78	4.19	4.17	3.74	3.57
ENN_MN	44.38	41.90	44.20	43.54	45.32	53.19
PLADJ	6.35	25.39	33.53	23.83	25.20	37.79
IJI	0.76	3.11	1.66	6.96	3.52	2.56
COHESION	0.24	11.11	16.36	20.03	11.51	26.18

As shown in Table 3, the landscape pattern indices of different landscape types had different sensitivity to the granularity changed. The set of class level indices with high sensitive to granularity change included NP, PD, ED, LPI, AREA_MN, ENN_MN, PLADJ, COHESION. The granularity effect analysis of the landscape pattern index of each landscape type in the high sensitivity showed that the suitable granularity range of cropland in the study area was 60–100 m, forestland 70–90 m, grassland 70–110 m, water body 80–100 m, built-up land 80–120 m and bare land 80–110 m. Based on the analysis results of granularity change of high sensitive landscape indices at the landscape level and class level, the optimal granularity range for landscape pattern analysis in this study area was finally determined to be 80–90 m.

**Figure 4:**
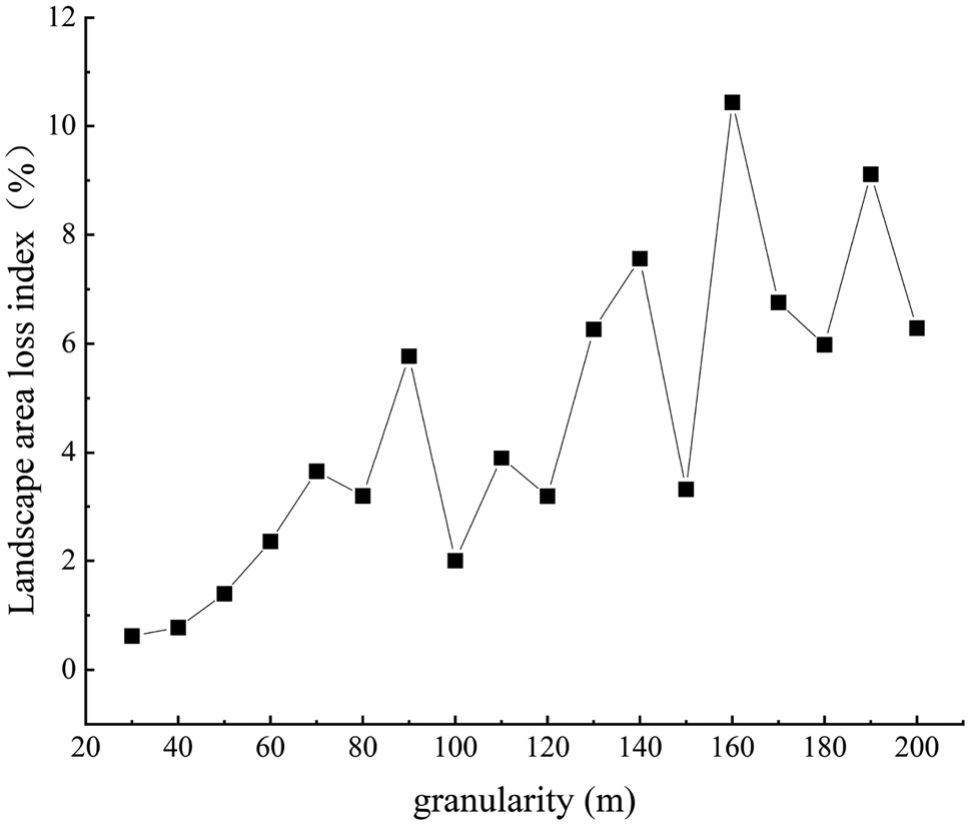
Accuracy loss of the landscape area under different granularities.

According to the calculation results of landscape area loss index in the study area under different granularities (Fig. 4), it was shown that when the granularity was less than 80 m, the landscape area loss index first increased and then decreased, and the overall change was not large, indicating that the impact of granularity changed on the landscape area loss was relatively small within this range. When the granularity was greater than 80 m, the fluctuation of landscape area loss index caused by granularity change increased, indicating that the granularity change had a greater impact on landscape area loss within this range, and the landscape area loss caused by granularity 90 m was larger than that caused by granularity 80 m. In order to ensure the calculation accuracy and the stability of the landscape pattern characteristics, 80 m was selected as the optimal granularity for spatial analysis of landscape pattern in this study area.

#### Amplitude effect analysis of landscape pattern index

1. Amplitude effect of PD.

**Figure 5:**
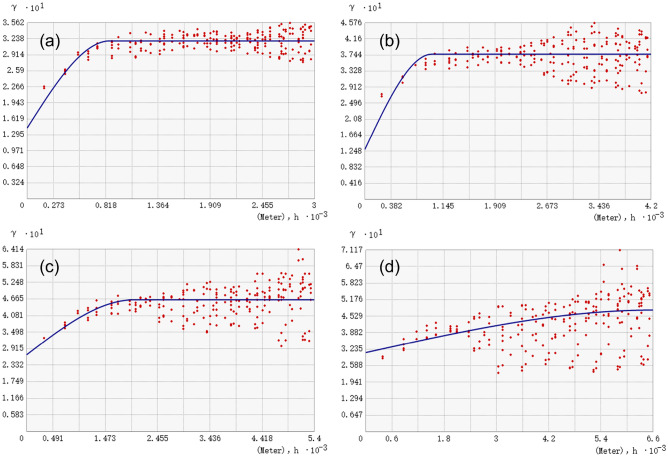
The semi-variogram fitting curves of PD under different amplitudes. (**a** 250 m, **b** 350 m, **c** 450 m, **d** 550 m).

Based on the results of the Granularity effects analysis, the land use maps were resampled into raster data with 80 m. According to the composition of the landscape pattern vulnerability index, the semi-variance function models of PD and SHDI at different amplitudes were fitted using AcrGIS10.6 software with the 2020 land use map as experimental data to determine the optimal amplitude for spatial variation in landscape pattern vulnerability. Spherical model was the most widely used theoretical model in geostatistical analysis. According to the global Moran’ I of PD and SHDI at different amplitudes, it could be seen that the Moran’ I index of the two ranges from 0.39 to 0.52 and 0.45 to 0.47, respectively, which mean that there was a positive spatial autocorrelation. Assuming that the landscape pattern index varied uniformly in space and there was no difference in direction, and the data structure in this study meet the spherical model fitting condition. Based on the principle of semi-variance model, the semi-variogram fitting curves of SHDI under different amplitudes are shown in Fig. 5.

As shown in Fig. 5, PD exhibited a significant spatial autocorrelation. The smaller the amplitude, the smaller the range of PD autocorrelation and the stronger the spatial autocorrelation. Within a certain distance, the degree of spatial variation of PD was positively correlated with the distance, and when the distance was small, the degree of spatial variation was relatively small. As the distance increases, the spatial autocorrelation weakens, the spatial difference of PD increased, and the semi-variogram increased. The semi-variogram at the 250 m and 350 m amplitudes could detect finer variations, and the sensitivity of the semi-variograms at the 250 m and 350 m amplitudes were significantly higher than that at 450 m and 550 m amplitudes.

The simulation results of the semi-variogram of PD under different amplitudes were shown in Table 4. Based on the theory of semi-variogram, the nugget should increase with the increase of amplitude, the degree of spatial variation caused by random factors should increase, and the nugget effect should also be enhanced. On the other hand, when the amplitude was smaller, the internal landscape type was simpler and the spatial autocorrelation of landscape index was stronger. However, when the amplitude was less than a certain range, it was believed that the original shape and structure of the landscape patch will be segmented and destroyed. As shown in Table 4, when the amplitude increased from 250 to 350 m, the nugget decreased from 0.144 to 0.129, which was inconsistent with the semi-variance function theory, indicating that the amplitude of 250 m was too small for the study area and destroyed the original landscape structure of the system. When the amplitude increased from 350 to 550 m, the nugget increased from 0.129 to 0.309, among them, when the amplitude increased to 450 m, the nugget increased the most, which was 0.13, indicating that the variation characteristics of smaller amplitude were covered up with the increased of amplitude, and the error was reflected in the nugget effect.

**Table 4:** The simulation results of the semi-variogram of PD under different amplitudes.

Amplitude/m	Nugget	Sill	Range	Nugget/sill
250	0.144	0.319	863	0.451
350	0.129	0.376	986	0.343
450	0.269	0.464	2022	0.580
550	0.309	0.477	6600	0.648

From the perspective of Nugget/sill, when the amplitude increased from 250 to 350 m, the Nugget/sill decreased from 0.451 to 0.343, indicating that the spatial variation degree of PD caused by random factors decreased with the increased of amplitude, while the spatial variation degree caused by spatial autocorrelation increased. This was inconsistent with the theory of semi-variogram, indicating that the 250 m amplitude was too small for the study area and damaged the original shape and structure of the landscape patch. When the amplitude increased from 350 to 550 m, the Nugget/sill increased from 0.343 to 0.648, indicating that as the amplitude increased, the contribution of spatial variation caused by random factors to the total spatial variation of PD increased, while the contribution of spatial variation caused by spatial autocorrelation to the total spatial variation decreased.

The range reflected the spatial distance of spatial autocorrelation of PD. When the amplitude was 250 m, the range was 863 m, indicating that the spatial autocorrelation of PD appeared in the range of 863 m under the amplitude of 250 m. When the range exceeded 863 m, there was no spatial autocorrelation. Meanwhile, we found that there was a significant positive correlation between the range and the amplitude. The larger the amplitude, the larger the local range of spatial autocorrelation. When the amplitude was 350 m, 450 m and 550 m respectively, there was no spatial autocorrelation when the range exceeded 986 m, 2022 m and 6600 m respectively. Overall, the amplitude of 250 m was too small, which was inconsistent with the theory of semi-variogram; The amplitudes of 450 m and 550 m were too large, which masked more local information. Therefore, the 350 m amplitude was the most suitable amplitude for analyzing the spatial variation of PD in this study area.

(2) Amplitude effect of SHDI.

**Figure 6:**
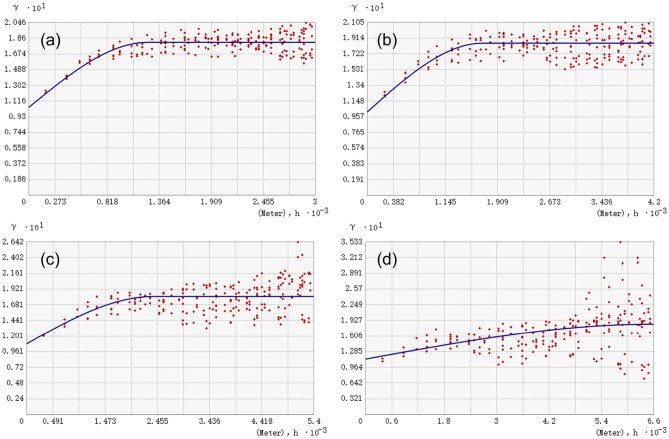
The semi-variogram fitting curves of SHDI under different amplitudes. (**a** 250 m, **b** 350 m, **c** 450 m, **d** 550 m).

As shown in Fig. 6, SHDI also had a significant spatial autocorrelation. Within a certain distance, the degree of spatial variation of SHDI was also positively correlated with the distance. When the distance increased, the spatial difference of SHDI also increased, and the semi-variation also increased. The semi-variogram at the 250 m and 350 m amplitudes could detect more subtle differences, while some of the detailed features of the semi-variants at the 450 m and 550 m amplitude were masked.

Similar to PD, the nugget decreased from 0.104 at 250 m amplitude to 0.102 at 350 m amplitude, indicating that the 250 m amplitude was too small for the study area and contained more local spatial information, which made the fitting results inconsistent with the semi-variogram theory (Table 5). Under the amplitude of 350 m to 550 m, the nugget increases with the increase of amplitude, indicating that the spatial heterogeneity of SHDI caused by random factors gradually increased, and the nugget effect was enhanced.

**Table 5:** The simulation results of the semi-variogram of SHDI under different amplitudes.

Amplitude/m	Nugget	Sill	Range	Nugget/sill
250	0.104	0.181	1244	0.575
350	0.102	0.185	1689	0.551
450	0.108	0.181	2388	0.597
550	0.113	0.185	6600	0.611

The change trend of Nugget / Sill of SHDI was also consistent with PD, when the amplitude increased from 250 to 350 m, Nugget / Sill decreased from 0.575 to 0.551, indicating that the overall spatial variability caused by random factors decreased, while the overall spatial variability caused by spatial autocorrelation increased. When the amplitude increased from 350 to 550 m, Nugget / Sill also showed an increased trend, indicating that the contribution of spatial variation caused by random factors to the total spatial variation gradually increased with the increase of amplitude, and the contribution of spatial variation caused by spatial autocorrelation to the total spatial variation gradually decreased with the increase of amplitude.

The range of SHDI was also positively correlated with the amplitude. When the amplitudes were 250 m, 350 m, 450 m and 550 m respectively, there was no spatial autocorrelation when the ranges exceeded 1244 m, 1689 m, 2388 m and 6600 m respectively, indicating that SHDI had spatial autocorrelation in a larger range. If the amplitude was too small, it was difficult to reflect the overall variation of regional variables, if the amplitude was too large, more local spatial information will be lost. Generally, the amplitude of 250 m was too small, which was inconsistent with the theory of semi-variogram. The amplitude of 450 m or 550 m was too large, and the description of the local spatial characteristics of SHDI was insufficient. The 350 m amplitude was also the most suitable amplitude for analyzing the spatial variation of SHDI in this study area. Therefore, it was determined that the optimal amplitude for analyzing the spatial variation characteristics of landscape pattern in this study area was 350 m.

### Evaluation of landscape pattern vulnerability

#### Spatial distribution of landscape pattern vulnerability

**Figure 7:**
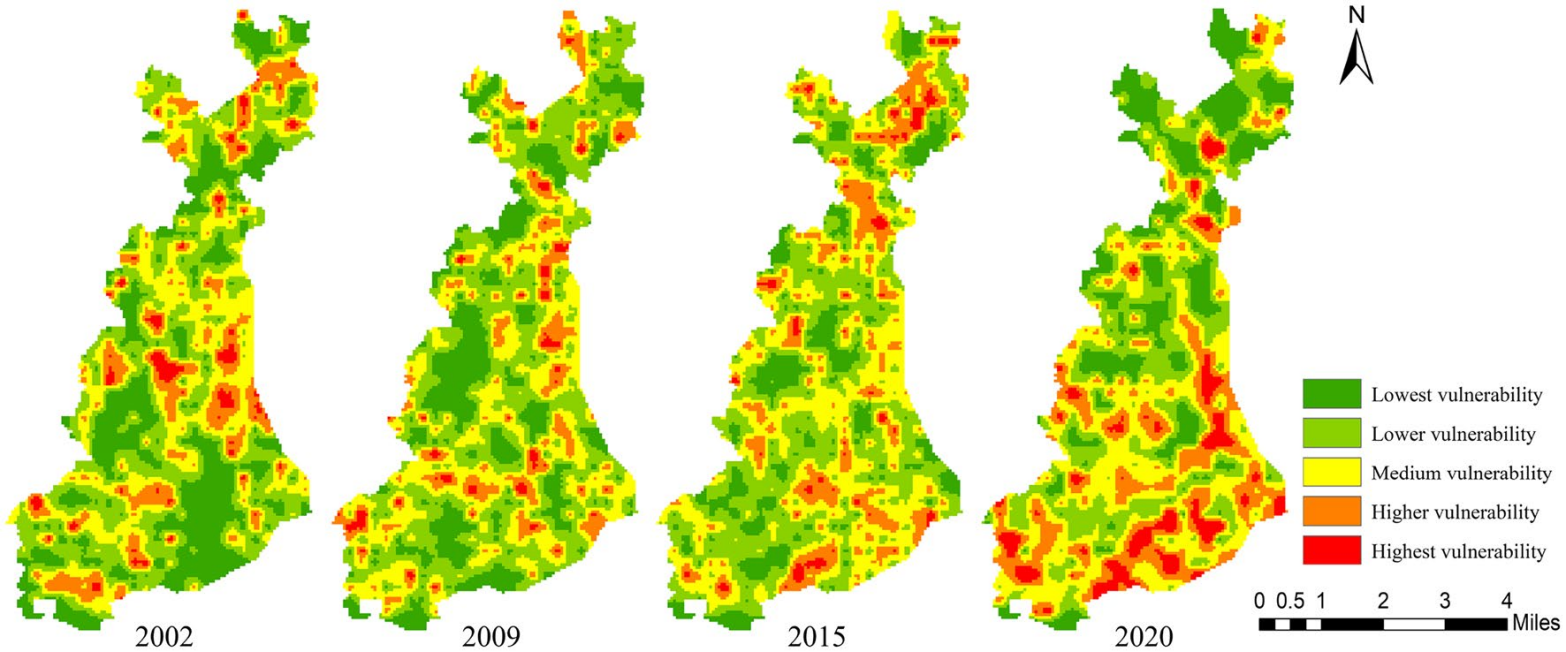
Spatial distribution of Landscape pattern vulnerability during 2002–2020. Cartographic software: ArcGIS online (https://www.esri.com/en-us/home).

Based on the results of semi-variance analysis, the study area was divided into 667 units at a amplitude of 350 × 350 m, and the LVI was calculated for each unit. The spatial distribution map of landscape pattern vulnerability in the study area from 2002 to 2020 was obtained by using the ordinary kriging interpolation tool in ArcGIS10.6 software (Fig. 7). In order to visually compare and analyze the spatial distribution characteristics of landscape pattern vulnerability in each period, the natural breaks classification method was used to uniformly divide the LVI into 5 levels: V-level highest vulnerability (LVI ≥ 0.2424), IV-level higher vulnerability (0.1946 ≤ LVI < 0.2424), III-level medium vulnerability (0.1548 ≤ LVI < 0.1946), II-level lower vulnerability (0.1136 ≤ LVI < 0.1548) and I-level lowest vulnerability (LVI < 0.1136). The mean values of LVI in 2002, 2009, 2015and 2020 were 0.1479, 0.1483, 0.1562 and 0.1625, respectively, showing an increasing trend year by year.

As shown in Fig. 7, there were significant differences in the spatial distribution of landscape pattern vulnerability in the study area. During the study period, the southern of the study area was greatly affected by human activities, and the expansion of built-up land inevitably occupied part of the surrounding cropland, forestland and grassland, resulting in a relatively high degree of landscape fragmentation. In 2020, the demolition of residential areas and road upgrading and reconstruction projects implemented by the government further increased the degree of landscape fragmentation in the southern region, while in the middle and northern regions, affected by land consolidation and reclamation policies, the land structure tended to be simplified and regularized, the degree of landscape fragmentation decreased, and the stability of landscape pattern increased. Combined with the land use distribution map in different periods, the spatial distribution characteristics of landscape pattern vulnerability in the study area was summarized as follows: The lower and lowest vulnerability areas were mainly consistent with the spatial distribution of cropland, water body and built up land with lower degree of fragmentation, higher connectivity and integrity. The degree of human disturbance in this area was relatively small and the self-recovery ability was relatively high. The higher and highest vulnerability areas were mainly consistent with the spatial distribution of grassland, forest land, built up land and some surrounding cropland with higher degree of fragmentation. Under the external interference, the land use change in this area was relatively frequent, and the stability of landscape structure was relatively poor. At the same time, in the concentrated distribution area of bare land, forestland and grassland, where the landscape dominance was higher and the adaptability of the landscape system to external interference was relatively low, resulting in a relatively high landscape pattern vulnerability in this region. The medium vulnerable areas were mainly distributed in the surrounding areas of higher and highest vulnerable areas. Although they had certain adaptability, the possibility of land use change under external disturbance was still relatively high.

#### Temporal evolution of landscape pattern vulnerability

**Figure 8:**
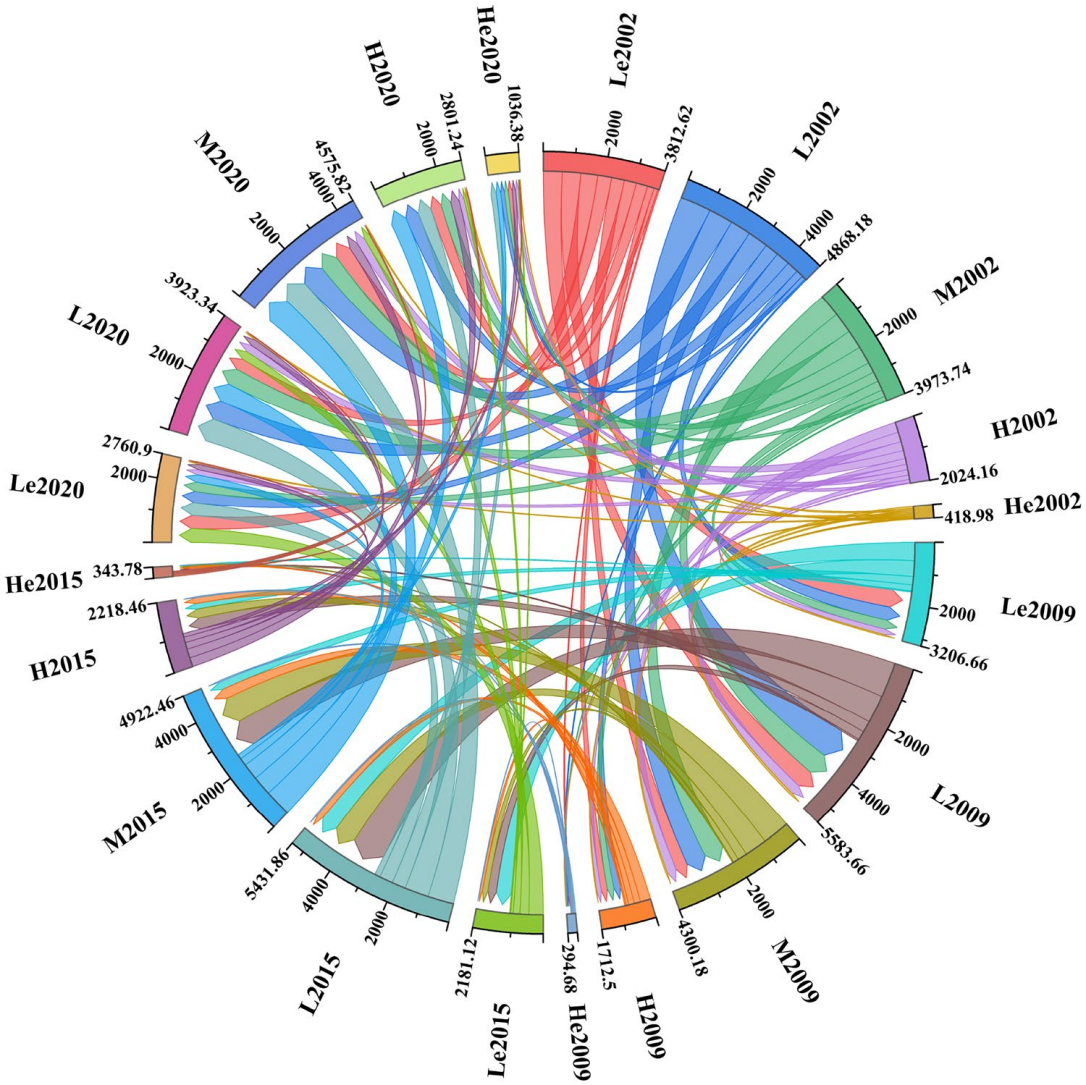
The transfer matrix of different levels of landscape pattern vulnerability during 2002–2020. (*Le*: Lowest vulnerability; *L*: Lower vulnerability; *M*: Medium vulnerability; *H*: Higher vulnerability; *He*: Highest vulnerability).

As shown in Tables 6, 7 and Fig. 8, the increase and decrease of landscape pattern vulnerable areas at different levels caused by land use change occur simultaneously. From 2002 to 2009, the areas of lowest, higher, and highest vulnerability decreased by 302.98 ha (4.01%), 155.83 ha (2.07%), and 62.15 ha (0.83%), respectively, while the area of lower and medium vulnerability increased by 357.74 ha (4.74%) and 163.22 ha (2.16%), respectively. The overall vulnerability of landscape pattern in the study area showed an increasing trend during this period, mainly showing that the lowest vulnerability areas shifted to lower and medium vulnerability areas.

**Table 6:** Statistics of different levels of landscape pattern vulnerability areas during 2002-2020

LVI level	2002		2009		2015		2020	
	Area (ha)	Proportion (%)	Area (ha)	Proportion (%)	Area (ha)	Proportion (%)	Area (ha)	Proportion (%)
Lowest vulnerability	1906.31	25.25	1603.33	21.24	1090.56	14.45	1380.45	18.29
Lower vulnerability	2434.09	32.24	2791.83	36.98	2715.93	35.98	1961.67	25.99
Medium vulnerability	1986.87	26.32	2150.09	28.48	2461.23	32.60	2287.91	30.31
Higher vulnerability	1012.08	13.41	856.25	11.34	1109.23	14.69	1400.62	18.55
Highest vulnerability	209.49	2.78	147.34	1.95	171.89	2.28	518.19	6.86

**Table 7:** Change of different levels of landscape pattern vulnerability areas during 2002–2020.

LVI level	2002–2009		2009–2015		2015–2020		2002–2020	
	Variety (ha)	Change percentage (%)	Variety (ha)	Change percentage (%)	Variety (ha)	Change percentage (%)	Variety (ha)	Change percentage (%)
Lowest vulnerability	− 302.98	− 4.01	− 512.77	− 6.79	289.89	3.84	− 525.86	− 6.96
Lower vulnerability	357.74	4.74	− 75.90	− 1.00	− 754.26	− 9.99	− 472.42	− 6.25
Medium vulnerability	163.22	2.16	311.14	4.12	− 173.32	− 2.29	301.04	3.99
Higher vulnerability	− 155.83	− 2.07	252.98	3.35	291.39	3.86	388.54	5.14
Highest vulnerability	− 62.15	0.83	24.55	0.33	346.30	4.58	308.70	4.08

From 2009 to 2015, the areas of lowest and lower vulnerability showed a decreasing trend, decreasing by 512.77 ha (6.79%) and 75.90 ha (1.00%), respectively, while the areas of medium, higher, and highest vulnerability showed an increasing trend, increasing by 311.14 ha (4.12%), 252.98 ha (3.35%), and 24.55 ha (0.33%), respectively. The overall vulnerability of landscape pattern in the study area showed an increasing trend during this period, mainly showing that the lowest vulnerability areas shifted to lower and highest vulnerability areas, and the lower vulnerability areas gradually shifted to medium and higher vulnerability areas.

From 2015 to 2020, the areas of lowest, higher and highest vulnerability showed an increasing trend, increasing by 289.89 ha (3.84%), 291.39 ha (3.86%) and 346.30 ha (4.58%), respectively, while the areas of lower and medium vulnerability showed a decreasing trend, decreasing by 754.26 ha (9.99%) and 173.32 ha (2.29%), respectively. The overall vulnerability of landscape pattern in the study area showed an increasing trend during this period, which was mainly manifested in the transfer of lower vulnerability areas to the other four types of vulnerability areas, the transfer of medium vulnerability areas to higher and highest vulnerability areas, and the transfer of higher vulnerability areas to lowest vulnerability areas.

Overall, during the whole study period, the areas of lowest and lower vulnerability showed a decreasing trend, decreasing by 525.86 ha (6.96%) and 472.42 ha (6.25%), respectively, while the areas of medium, higher, and highest vulnerability showed an increasing trend, increasing by 301.04 ha (3.99%), 388.54 ha (5.14%), and 308.70 ha (4.08%), respectively. The overall vulnerability of the landscape pattern in the study area showed an increasing trend, mainly showing that the lowest and lower vulnerability areas shifted to medium and higher vulnerability areas, and the lowest and medium vulnerability areas shifted to highest vulnerability areas.

**Figure 9:**
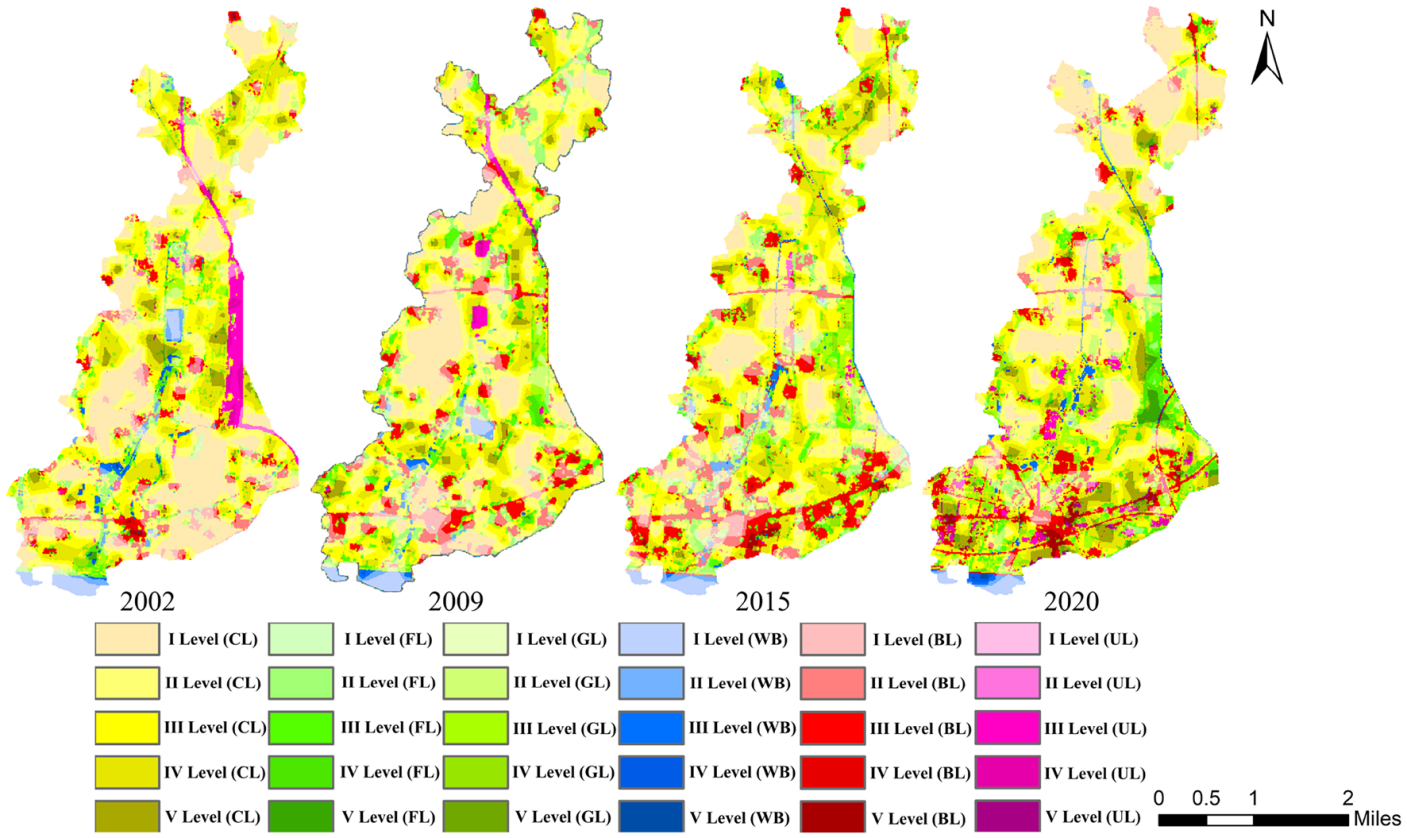
Spatial distribution of vulnerability of each landscape types during 2002–2020. Cartographic software: ArcGIS online (https://www.esri.com/en-us/home). (*CL*: Cropland; *FL*: Forestland; *GL*: Grassland; *WB*: Water body; *BL*: Built up land; *UL*: Bare land).

**Figure 10:**
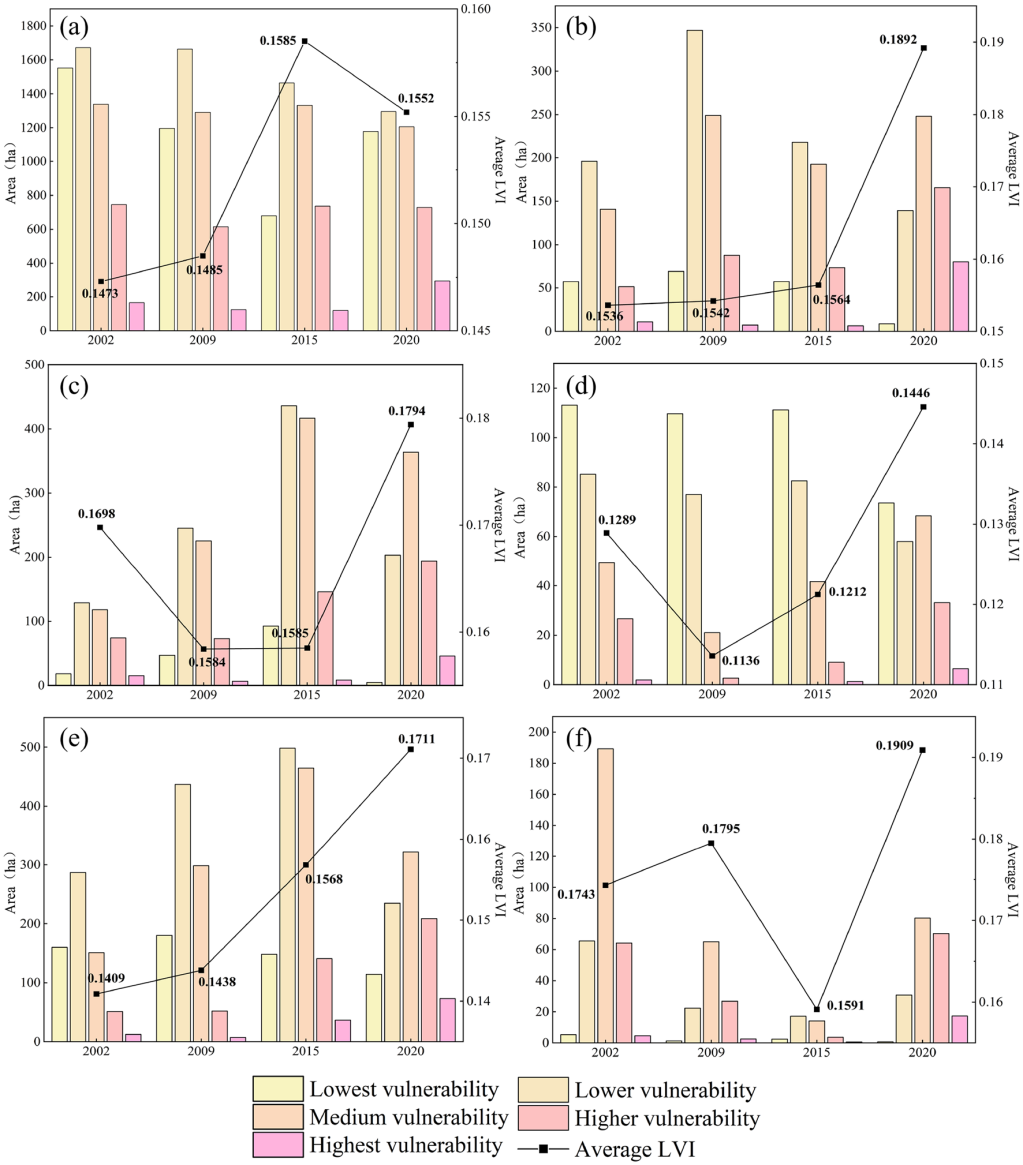
Vulnerability area and average vulnerability index of each landscape type during 2002–2020 (**a**: Cropland; **b**: Forestland; **c**: Grassland; **d**: Water body; **e**: Built up land; **f**: Bare land).

As shown in Figs. 9 and 10, in terms of land use, during the whole period, the overall vulnerability of the six landscape types showed an increasing trend. For cropland, the area in the V level vulnerability increased by 130.46 ha, while in the I level, II level, III level and IV level vulnerability showed a decreasing trend, with a decrease of 373.58 ha, 376.22 ha, 132.44 ha, and 15.85 ha, respectively. The average landscape vulnerability index of cropland increased by 5.36%. For forestland, the area in the I level and II level vulnerabilities decreased by 48.85 ha and 56.62 ha, respectively, while in the III level, IV level and V level vulnerabilities increased by 107.27 ha, 114.25 ha, and 69.63 ha, respectively. The average landscape vulnerability index of forestland significantly increased, with an increase of 23.18%. For grassland, the area in the I level vulnerability was relatively small and slightly reduced, while in the other four levels vulnerabilities showed an increasing trend. The average landscape vulnerability index of grassland increased by 5.65%. For water body, the area in the I level and II level vulnerabilities decreased by 39.55 ha and 27.22 ha respectively, while in the III level, IV level and V level showed an increasing trend, with an increase of 18.96 ha, 6.55 ha, and 4.56 ha respectively. The average landscape vulnerability index of water body increased by 12.18%. For built up land, the area in the I level and II level vulnerabilities decreased by 45.76 ha and 51.97 ha, respectively, while in the III level, IV level and V level increased by 170.70 ha, 157.62 ha, and 60.65 ha, respectively. The average landscape vulnerability index of built up land significantly increased, with an increase of 21.43%. For bare land, the area in the IV level and V level vulnerabilities showed an increasing trend, increasing by 6.05 ha and 12.71 ha, respectively, while in the I level, II level and III level vulnerabilities decreased by 4.01 ha, 34.76 ha and 108.97 ha, respectively. The average landscape vulnerability index of bare land increased by 9.52%.

### Spatial auto‑correlation of landscape pattern vulnerability

#### Global spatial auto‑correlation analysis

**Figure 11:**
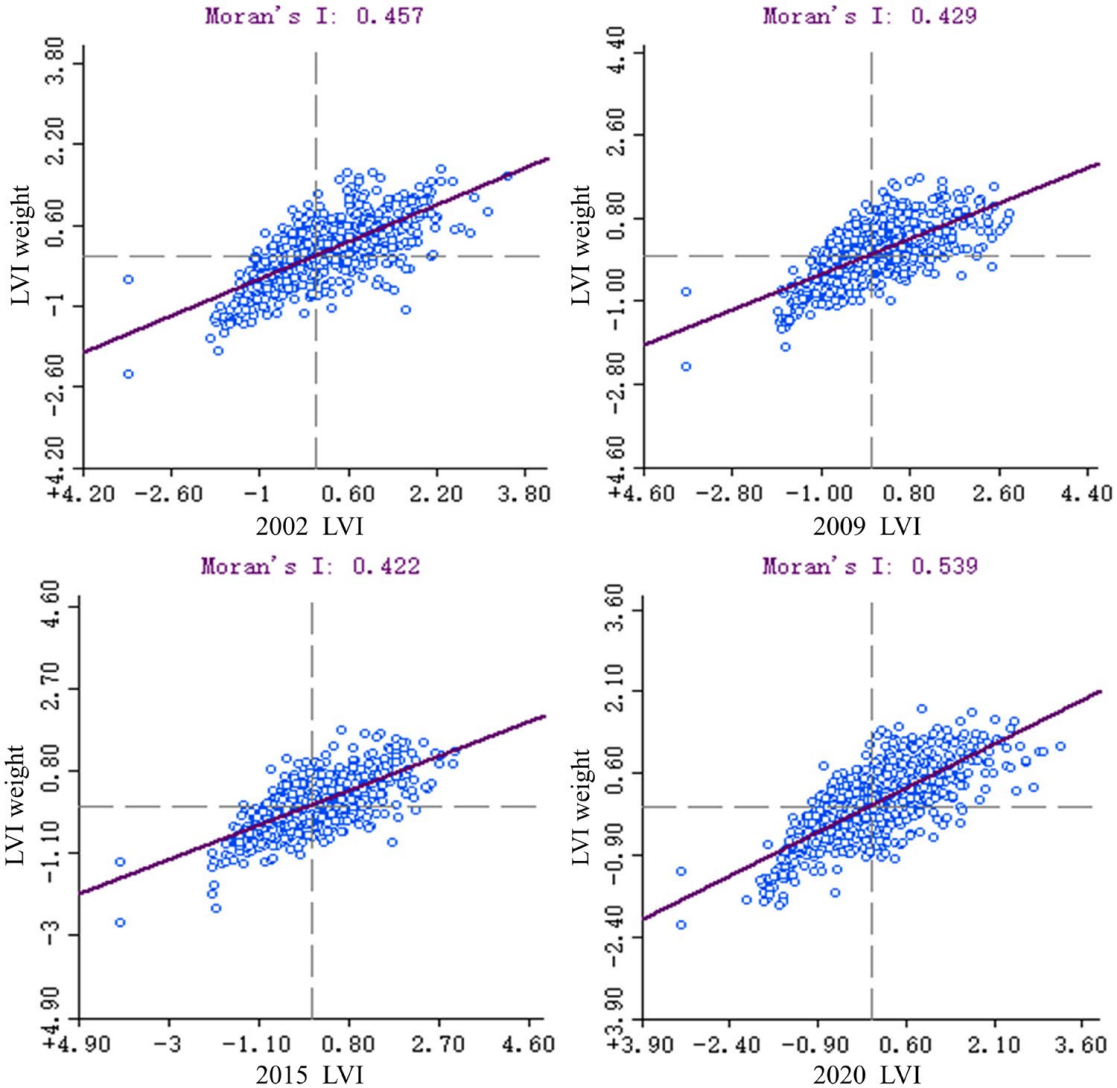
Moran scatter plots of landscape pattern vulnerability during 2002–2020.

In order to further explore the spatial distribution characteristics of landscape pattern vulnerability in the study area, the Moran scatter plots are used to analyze the spatial correlation of the landscape pattern vulnerability (Fig. 11). The global Moran’s I values in the four years were 0.457,0.429,0.422 and 0.539, respectively, and the scatter points were mainly distributed in the first and third quadrants, indicating that the landscape pattern vulnerability of the four periods in the study area had a significant spatial positive correlation, and the distribution pattern showed a spatial agglomeration characteristics. However, the spatial aggregation of landscape pattern vulnerability decreased from 2002 to 2015, and increased after 2015. Overall, the degree of the spatial agglomeration of the landscape pattern vulnerability showed an increasing trend.

#### Local spatial auto‑correlation analysis

**Figure 12:**
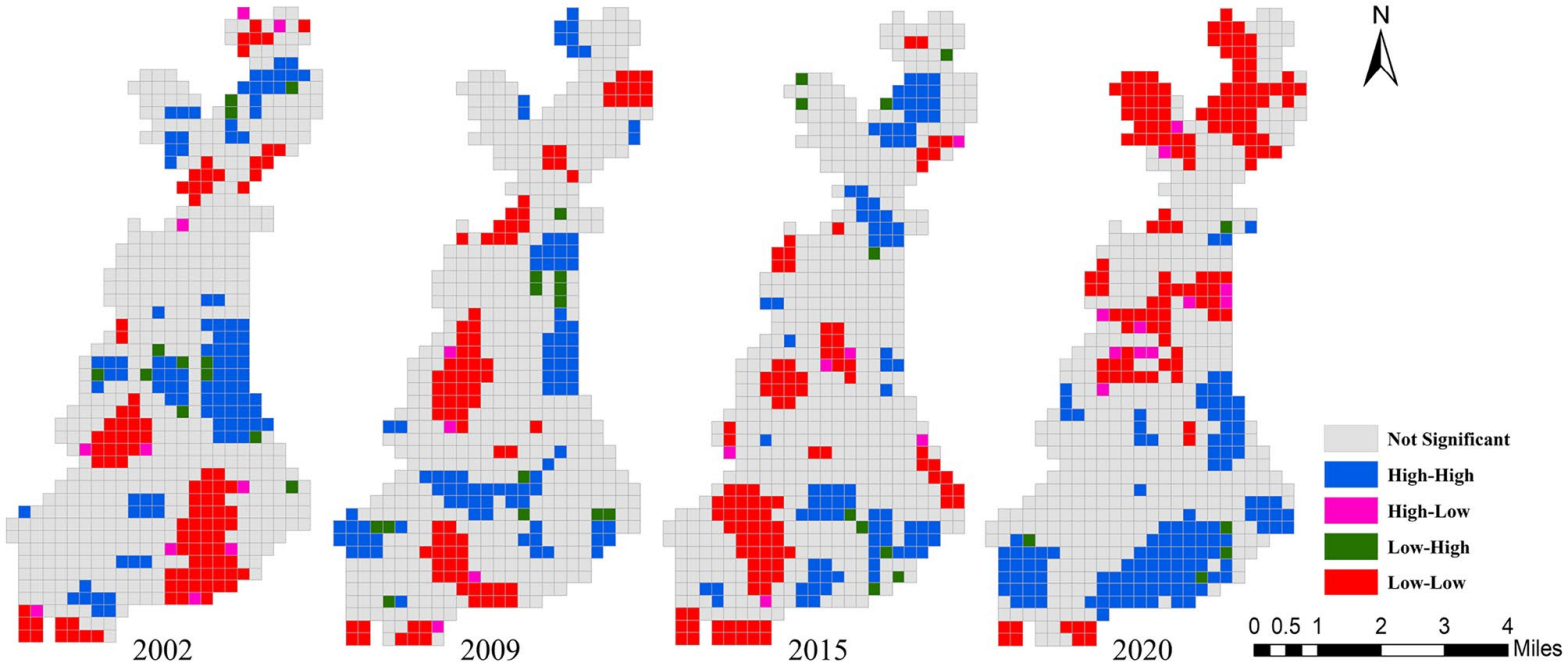
LISA cluster map of landscape pattern vulnerability during 2002–2020. Cartographic software: Geoda online (https://geodacenter.github.io).

In order to clarify where the spatial agglomeration was and why the global Moran’s I decreased first and then increased from 2002 to 2020, LISA cluster maps were used to visualize the local clustering characteristics of landscape pattern vulnerability (Fig. 12). Significant auto-correlations were grouped into four types: high–high (HH), low–low (LL), low–high (LH), and high–low (HL).

**Figure 13:**
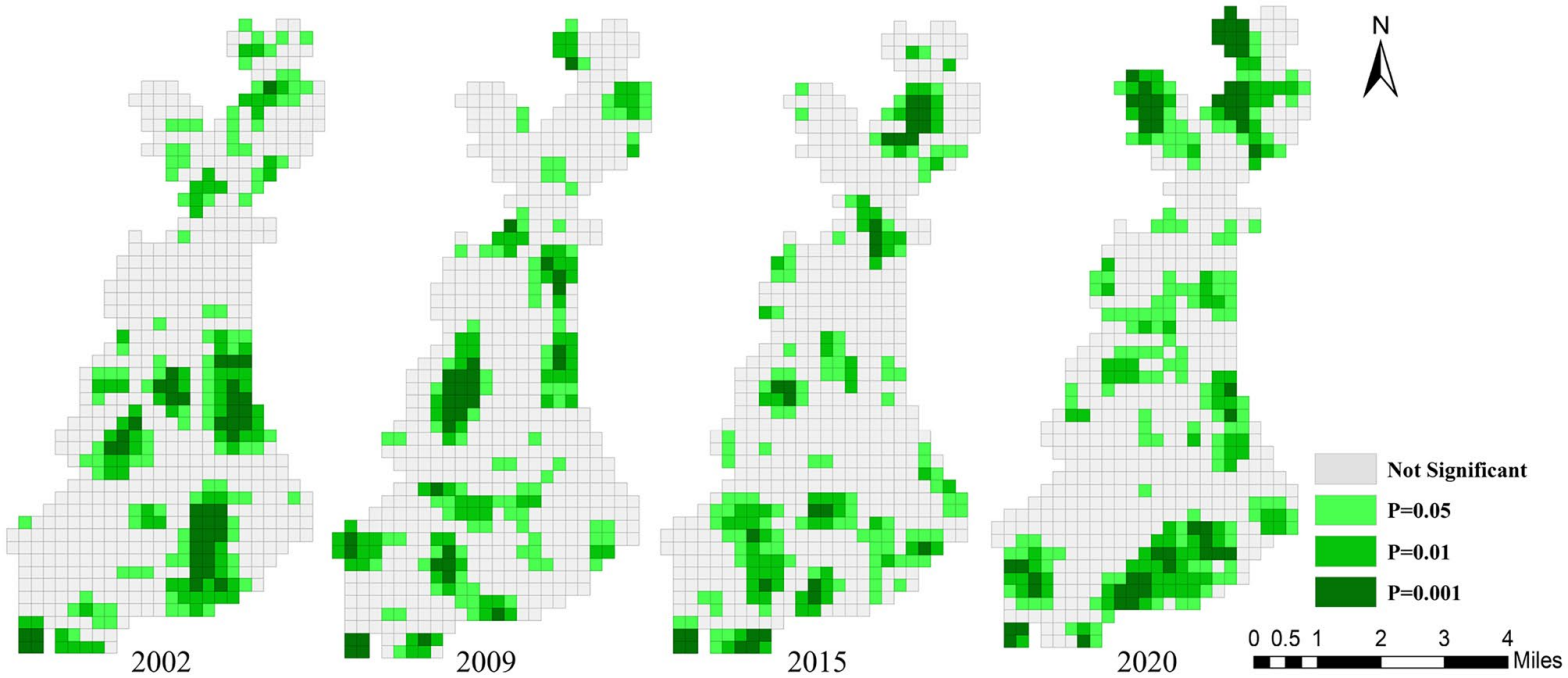
LISA significance map during 2002–2020. Cartographic software: Geoda online (https://geodacenter.github.io).

As shown in Fig. 12, the spatial distribution of local aggregation of landscape pattern vulnerability is consistent with the spatial interpolation results of LVI. During 2002–2020, the HH and LL areas increased from 14.84% and 14.69% to 18.14% and 19.49%, respectively. From the spatial distribution, the spatial aggregation of the two has increased significantly. The HH areas were generally transferred from the middle to the southern. This is mainly due to that the southern of the study area is a key development region in the middle of the startup area in the future. In order to introduce high-tech industries, promote energy conservation and emission reduction, and improve residents’ quality of life, some old residential areas and primary and secondary roads have been renovated since 2019, resulting in a relatively high degree of landscape fragmentation in the southern of the study area. The LL areas were generally transferred from the southeastern and midwestern to the middle and northern, which mainly benefited from the impact of returning farmland to forest/grass and reclamation policies. The spatial distribution of forestland, grassland and cropland in these areas tended to be aggregated, simple and regular from dispersion, fragmentation and complexity. The degree of landscape fragmentation was reduced, the integrity and connectivity of patches were improved, and the anti-interference ability was enhanced. As can be seen from Fig. 13, the spatial aggregation of most HH and LL areas in the study area reached 0.05 or 0.01 significance level, and some areas even reached 0.001 significance level.

## Discussion

Spatial heterogeneity was the basic feature of landscape pattern, and there were significantly differences with the change of scales^35^. The optimal landscape scale could not only be used to accurately explored the temporal and spatial variation characteristics of landscape pattern, but also scientifically revealed the change laws of landscape ecological structure and function^40^. However, when scholars conducted research on the landscape pattern vulnerability, they mostly focus on exploring the laws of spatiotemporal evolution, ignoring the impact of scale selection on the spatial heterogeneity of landscape pattern vulnerability^40^. This has a negative impact on the scientificity and accuracy of the assessment results, and hinders the sustainable development of human society to a certain extent. Therefore, this paper used the coefficient of variation method, the granularity effect curve and the information loss model to determine the optimal granularity of the study area at the landscape and class level as 80 m. At the same time, the grid method and semi variance function were used to determine the optimal amplitude of the study area as 350 × 350 m. These methods had been widely verified in landscape pattern studies of many river basins in China. However, there were significant differences in the results for different regions. The optimal grid scales for landscape pattern research in the Bailong river basin and the Yellow river basin were 5 × 5 km and 90 × 90 km, respectively^54,55^. The optimal granularity and amplitude of landscape ecological risk assessment in Shiyang river basin were 60 m and 4.5 × 4.5 km, respectively^56^. Then, on the basis of the determined optimal scale, the landscape pattern vulnerability evaluation model was constructed by using LSI and LAI, and the spatial and temporal evolution law and spatial aggregation characteristics of landscape pattern vulnerability in the study area were discussed. During 2002–2020, the spatial distribution characteristics of the landscape pattern vulnerability showed that the southern of the study area was greatly affected by human disturbance, the degree of fragmentation and the vulnerability of landscape pattern increased. In the middle and northern, the stability of landscape pattern was enhanced due to the influence of land consolidation and reclamation policies. The research results are consistent with the actual ecological environmental status of the study area. As a key development region in the middle of the startup area in the future, the landscape pattern changes in the southern of the study area will be more complex, and the landscape pattern vulnerability may be further increased^41^. Special attention should be paid to the negative impact of fragmented landscape patterns on the ecological environment. Meanwhile, in order to encourage agricultural production and maintain food security, a modern agricultural industry system will be established in the middle and northern of the study area^41^. The land structure will be simpler and more regular, and the landscape connectivity and integrity will be further increased. The landscape pattern vulnerability in these regions may be further decreased. This study provides a new case for selecting an optimal scale of analysis for landscape pattern vulnerability, and is of great significance for obtaining more convincing assessment results and promoting the sustainable planning and management of landscape pattern. However, there are still some limitations and uncertainties that need to be improved. In this study, the selection of the optimal scale and the vulnerability analysis of landscape pattern were based on the landscape pattern index. The accuracy of land use classification directly affects the calculation results of landscape pattern index^57,58^. Therefore, improving the accuracy of land use classification needs further research. Researches have shown that natural and socio-economic factors have an impact on landscape pattern vulnerability. In order to facilitate decision makers to better implement targeted ecosystem protection measures, the driving factors of landscape pattern vulnerability need further explore.

## Conclusion

This study used Landsat satellite images from 2002, 2009, 2015, and 2020 to analyze the spatiotemporal evolution characteristics and spatial autocorrelation of landscape pattern vulnerability in Dasi river basin in the startup area, based on determining the optimal granularity and amplitude of landscape pattern. The main conclusions of this study are as follows:

1. The optimal granularity and amplitude for landscape pattern research in this study area were 80 m and 350 × 350 m, respectively.
2. During 2002–2020, the fragmentation degree and vulnerability of landscape pattern in the southern of the study area increased, while the stability of landscape pattern in the middle and northern enhanced.
3. The overall vulnerability of the landscape pattern showed an increasing trend, which was mainly manifested in the transfer of lowest and lower vulnerability areas shifted to medium and higher vulnerability areas, and the lowest and medium vulnerability areas shifted to highest vulnerability areas. From the perspective of land use, the average vulnerability of the six landscape types showed an increasing trend. Among them, the average vulnerability indices of forestland and built up land changed the most, increased by 23.18% and 21.43%, respectively, followed by water body and bare land, increased by 12.18% and 9.52%, respectively, and cropland and grassland were relatively small, increased by 5.36% and 5.65%, respectively.
4. The landscape pattern vulnerability in the study area showed a significant spatial positive correlation and high spatial clustering. During 2002–2020, the degree of the overall spatial agglomeration of the landscape pattern vulnerability showed an increasing trend. Among, the LL areas were generally transferred from the southeastern and midwestern to the middle and northern, the HH areas were mainly transferred from the middle to the southern.
5. In the future, the landscape pattern vulnerability in the southern of the study area may be further increased. While the landscape pattern vulnerability in the central and northern may be further decreased.

## Data Availability

The datasets generated or analyzed during this study are available upon request from the corresponding author.
